# Distribution and molecular drivers of the 21-gene recurrence score in early breast cancer with low, intermediate, and high estrogen receptor expression

**DOI:** 10.3389/fimmu.2026.1653477

**Published:** 2026-04-30

**Authors:** Shuai Li, Jiahui Huang, Yifei Zhu, Jin Hong, Siji Zhu, Weiqi Gao, Ou Huang, Jianrong He, Weiguo Chen, Yafen Li, Xiaosong Chen, Kunwei Shen, Jiayi Wu

**Affiliations:** Department of General Surgery, Comprehensive Breast Health Center, Ruijin Hospital, Shanghai Jiao Tong University School of Medicine, Shanghai, China

**Keywords:** 21-gene recurrence score, breast cancer, ER-low, HER2-negative, molecular drivers

## Abstract

**Background:**

Estrogen receptor (ER)-low human epidermal growth factor receptor 2 (HER2)-negative breast cancer shares similar pathological and molecular features with triple-negative breast cancer, yet the utility of the 21-gene assay in this population remains uncertain. This study aimed to evaluate the distribution and molecular drivers of the 21-gene recurrence score (RS) in early breast cancer with low, intermediate, and high ER expression.

**Methods:**

ER-positive, HER2-negative early breast cancer patients with 21-gene testing results from Shanghai Jiao Tong University Breast Cancer Database (2009-2022) were included. Eligible patients were categorized into three groups: ER-low (1-9%), ER-intermediate (10-50%), and ER-high (>50%). Chi-square and Kruskal-Wallis tests were conducted to compare the 21-gene RS and expression profiles among these groups. Spearman correlation and linear variance decomposition were performed to assess the associations between RS and its constituent gene modules.

**Results:**

A total of 4754 patients were included (ER‑low: 70, 1.5%; ER‑intermediate: 129, 2.7%; ER‑high: 4555, 95.8%). Mean RS values were 39, 32, and 24 in the three groups, with RS >25 observed in 72.9%, 62.8%, and 42.5% of patients, respectively (P < 0.001).. Among patients with ER-low tumors, the rates of RS >25 were 62.5% and 81.6% for those ≤50 or >50 years, 66.7% and 83.3% for those with low or high clinical risk, and 61.6% and 87.1% for those with PR-positive or PR-negative diseases, respectively. Notably, 90.6% younger patients had RS >15 in the ER-low group. Expression of *ESR1* (*P* < 0.001), *PGR* (*P* < 0.001), *BCL2* (*P* = 0.035), *SCUBE2* (*P* < 0.001) from the ER module, and *STMY3* (*P* = 0.001) from the invasion module were significantly lower in ER-low than in ER-high. Only, the proliferation module had a modest correlation with RS among patients with ER-low tumors (R = 0.34, *P* = 0.0076). Variance decomposition showed that the four modules explained only 8.83% of RS variance in ER‑low tumors (7.38% from proliferation), compared with 48.74% (42.93% from ER module) in ER‑high and 28.97% (21.94% from ER module) in ER‑intermediate tumors.

**Conclusions:**

The 21-gene RS was higher in ER-low breast cancer patients, particularly among those ≤50 years, with high clinical risk or PR-negative diseases. RS was only moderately correlated with proliferation features and minimally determined by any of the four module components in ER-low tumors. These findings are exploratory and require further validation in larger cohorts.

## Introduction

Estrogen receptor (ER)-low breast cancer, defined as tumors with 1-9% ER expression by immunohistochemistry, represents a unique and clinically challenging subgroup within the spectrum of ER-positive breast cancers (1). These tumors share several pathological and molecular features with triple-negative breast cancer (TNBC), including higher proliferation rates (2–4), lower expression of ER-related genes (5, 6), and a more aggressive clinical behavior compared to ER-high tumors (>50%) (7, 8). Despite being classified as ER-positive, ER-low tumors often exhibit limited response to endocrine therapy (9–12) and a higher risk of recurrence (13), highlighting the need for refined prognostic and therapeutic strategies in this population.

The 21-gene Recurrence Score (RS) assay has become a cornerstone in the management of ER-positive, HER2-negative early breast cancer (14–17). By evaluating the expression of 16 cancer-related genes and five reference genes, the assay provides a composite score that stratifies patients into low-, intermediate-, and high-risk categories (18, 19), thereby guiding adjuvant treatment decisions (20–24). However, the applicability and clinical utility of the 21-gene assay in ER-low tumors remain controversial. Given the molecular similarities between ER-low and TNBC, it is unclear whether the 21-gene assay can effectively predict recurrence risk or provide meaningful therapeutic guidance in this specific subgroup.

In this study, we first compared the distribution of 21-gene RS among patients with different ER levels. Specifically, we explored the clinical implications of RS in ER-low patients based on age, clinical risk, and PR status. Furthermore, we evaluated the gene expression profiles and molecular drivers of RS in ER-low tumors. We aimed to provide insights into the appropriate use of the 21-gene assay in this challenging ER-low subgroup of breast cancer patients.

## Materials and methods

### Patients

Consecutive patients who underwent surgery for invasive breast cancer at the Department of General Surgery, Comprehensive Breast Health Center, Ruijin Hospital from January 2009 to December 2022 were retrospectively screened. Clinicopathological features, adjuvant treatments and follow-up data were retrieved from Shanghai Jiao Tong University Breast Cancer Database (SJTU-BCDB). The main entry criteria were as follows: (1) ER-positive (≥1% nuclear staining by IHC); (2) HER2-negative (IHC -/1+ or IHC 2+ with non-amplification by ISH); (3) no distant metastasis at the time of diagnosis; (4) 21-gene RS results. IHC assessments of ER were performed using SP1 (DAKO) and Ventana Autostain System (BenchMark XT) in the Department of Pathology, Ruijin Hospital. ER expression was quantified as the percentage of tumor cells with nuclear staining. Tumors with 1-9% staining were classified as ER-low, 10-50% as ER-intermediate, and >50% as ER-high. Clinical risk was defined according to the MINDACT trial criteria: patients with tumors of (1) ≤3 cm and Grade I, (2) ≤2 cm and Grade II, (3) ≤1 cm and Grade III were classified as clinical low-risk while others were considered clinical high-risk (25). In cases with lymph node involvements, only those with tumor size ≤2 cm and Grade I were categorized as clinical low-risk. The present study was reviewed and approved by independent ethics committees of Ruijin Hospital, and the research met the requirements for the protection of patients.

### The 21-gene RS assay

The reverse transcription polymerase chain reaction (RT-PCR) assay of 21 genes was performed on formalin-fixed, paraffin-embedded (FFPE) tissue by the Department of Pathology, Ruijin Hospital as described previously (26). In brief, hematoxylin and eosin-stained slide was firstly reviewed to ensure sufficient invasive breast cancer in the tissue. Subsequently, total RNA was extracted and purified from three 10-μm unstained sections using the RNeasy FFPE RNA kit (Qiagen, 73504, Germany). Gene-specific reverse transcription was conducted using Omniscript RT kit (Qiagen, 205111, Germany). Finally, quantitative RT-PCR was performed in 96-well plates with Applied Biosystems (Foster City, CA, USA) 7500 Real-Time PCR system. Expression of each gene was measured in triplicate, and normalized relative to a set of five reference genes. The RS, ranging from 0 to100, was derived from the reference-normalized expression measurements for the 16 cancer-related genes. The genes were grouped based on gene function and correlated expression. ER group genes included *ESR1*, *PGR*, *BCL2*, and *SCUBE2*. HER2 group genes included *GRB7* and *ERBB2*. Invasion group genes included *STMY3* and *CTSL2*. Proliferation group genes included *MKI67*, *STK15*, *BIRC5*, *CCNB1*, and *MYBL2*. Module scores of RS were derived from reference-normalized expression of module genes using a non-linear algorithm.

### Treatment and follow-up

Adjuvant therapy decisions were decided by our multi-disciplinary team according to guidelines such as ASCO, NCCN. Patients were followed up at the out-patient department every 3 months in the first two years after surgery, every 6 months between the 3^rd^ and 5^th^ years, and once every year thereafter. Disease-free survival (DFS) was calculated from the date of surgery to the first date of local-regional recurrence, contralateral breast cancer, secondary primary malignancy, distant metastasis or death. Overall survival (OS) was defined as the time until death from any cause. The last follow-up date was July 5 2024.

### Statistical analysis

Clinicopathological characteristics including the categorical 21-gene RS of the three groups were compared via Chi-square test or Fisher exact test. Nonparametric Kruskal-Wallis tests were conducted to compare the continuous 21-gene RS and gene expressing profiles among these groups. To assess the association between molecular features and the RS score, Spearman correlation and linear variance analyses were performed on the RS score and its constituent modules as surrogates. Generally, a Spearman correlation coefficient >0.8 was considered strong, 0.3-0.8 moderate, and <0.3 weak. Rates of DFS and OS were estimated from Kaplan-Meier curves and compared via Log-rank test among the three groups. Cox proportional hazards models were used to investigate independent prognostic factors as well as to calculate hazard ratios (HRs) and 95% confidence intervals (CIs). *P* < 0.05 was considered statistically significant. All statistical procedures were performed with R 3.6.2 software (R Foundation for Statistical Computing).

### Data availability statement

The datasets used and/or analyzed during the current study are available from the corresponding author on reasonable request.

## Results

### Patients and tumor characteristics

A total of 4754 patients were included. Detailed patients and tumor characteristics are summarized in **Table 1**. The median age at the time of diagnosis was 58 years, with 33.5% being 50 years old or younger. Clinically, 2398 patients (50.4%) were classified as low-risk, while 2004 (42.2%) were deemed high-risk. The mean 21-gene RS was 24 (IQR 19-30), with 5.1% <11, 51.4% between 11 and 25, and 43.5% >25.

**Table 1:** Baseline clinical and pathological characteristics by ER level.

Characteristics	Total 4754 (%)	ER-high 4555 (%)	ER-intermediate 129 (%)	ER-low 70 (%)	*P* value
Age (y/o)					< 0.001
Mean (IQR)	58 (47-66)	58 (47-67)	52 (45-61)	52 (44-60)	
≤50	1574 (33.1)	1487 (32.6)	55 (42.6)	32 (45.7)	
>50	3180 (66.9)	3068 (67.4)	74 (57.4)	38 (54.3)	
Breast surgery					0.004
Mastectomy	2490 (52.4)	2363 (51.9)	82 (63.6)	45 (64.3)	
BCS	2264 (47.6)	2192 (48.1)	47 (36.4)	25 (35.7)	
Histology type					0.693
IDC	3934 (82.8)	3765 (82.7)	109 (84.5)	60 (85.7)	
Non-IDC	820 (17.2)	790 (17.3)	20 (15.5)	10 (14.3)	
Tumor size					0.052
≤2.0 cm	3218 (67.7)	3091 (67.9)	89 (69.0)	38 (54.3)	
>2.0 cm	1536 (32.3)	1464 (32.1)	40 (31.0)	32 (45.7)	
ALN status					0.024
Negative	3995 (84.0)	3814 (83.7)	117 (90.7)	64 (91.4)	
Positive	759 (16.0)	741 (16.3)	12 (9.3)	6 (8.6)	
Histological grade					< 0.001
I	445 (9.4)	431 (9.5)	13 (10.0)	1 (1.4)	
II	2934 (61.7)	2843 (62.4)	61 (47.3)	30 (42.9)	
III	819 (17.2)	757 (16.6)	37 (28.7)	25 (35.7)	
NA	556 (11.7)	524 (11.5)	18 (14.0)	14 (20.0)	
Clinical risk					0.465
Low	2398 (50.4)	2300 (50.5)	67 (51.9)	31 (44.3)	
High	2202 (42.2)	1922 (42.2)	52 (40.3)	30 (42.9)	
NA	352 (7.4)	333 (7.3)	10 (7.8)	9 (12.8)	
PR status					< 0.001
Positive	4296 (90.4)	4166 (91.5)	84 (65.1)	39 (55.7)	
Negative	458 (9.6)	389 (8.5)	45 (34.9)	31 (44.3)	
HER2 status					0.223
-	1011 (21.3)	959 (21.1)	33 (25.6)	19 (27.1)	
+/++	3743 (78.7)	3596 (78.9)	96 (74.4)	51 (72.9)	
Ki-67 level					0.040
<20%	2718 (57.2)	2620 (57.5)	67 (51.9)	31 (44.3)	
≥20%	2036 (42.8)	1935 (42.5)	62 (48.1)	39 (55.7)	
21-gene RS					< 0.001
Mean (IQR)	24 (19-30)	24 (19-30)	32 (21-44)	39 (24-56)	
<11	243 (5.1)	235 (5.2)	5 (3.9)	3 (4.2)	
11-25	2442 (51.4)	2383 (52.3)	43 (33.3)	16 (22.9)	
>25	2069 (43.5)	1937 (42.5)	81 (62.8)	51 (72.9)	
Chemotherapy					< 0.001
No	2620 (55.1)	2546 (55.9)	56 (43.4)	18 (25.7)	
Yes	2134 (44.9)	2009 (44.1)	73 (56.6)	52 (74.3)	
Endocrine therapy					< 0.001
No	7 (0.1)	0 (0.0)	0 (0.0)	7 (10.0)	
Yes	4747 (99.9)	4555 (100.0)	129 (100.0)	63 (90.0)	
Radiotherapy					< 0.001
No	2316 (48.7)	2192 (48.1)	82 (63.6)	42 (60.0)	
Yes	2438 (51.3)	2363 (51.9)	47 (36.4)	28 (40.0)	

ALN, axillary lymph node; ALND, ALN dissection; BCS, breast-conserving surgery; ER, estrogen receptor; HER2, human epidermal growth factor receptor-2; IDC, invasive ductal carcinoma; IQR: interquartile range; NA, not available; PR, progesterone receptor; RS: recurrence score; y/o, years old.

The significant *P* values are in bold.

Among the study cohort, 4555 patients (95.8%) had ER-high tumors, 129 (2.7%) had ER-intermediate tumors, and 70 (1.5%) had ER-low tumors (**Table 1**). Patients with ER-low and ER-intermediate tumors were significantly younger compared to those with ER-high tumors (52 *vs.* 52 *vs.* 58 years, *P* < 0.001). With ER level decreasing, the proportions of grade III tumors (16.6% *vs.* 38.7% *vs.* 35.7%, *P* < 0.001), PR-negativity (8.5% *vs.* 34.9% *vs.* 44.3%, *P* < 0.001), and Ki67 ≥ 20% (42.5% *vs.* 48.1% *vs.* 55.7%, *P* = 0.040) increased significantly. Furthermore, the rate of chemotherapy administration was significantly higher in the ER-low and ER-intermediate groups compared to the ER-high group (74.3% *vs.* 56.6% *vs.* 44.1%, *P* < 0.001).

### Distribution of 21-gene RS by ER level

**Figure 1:**
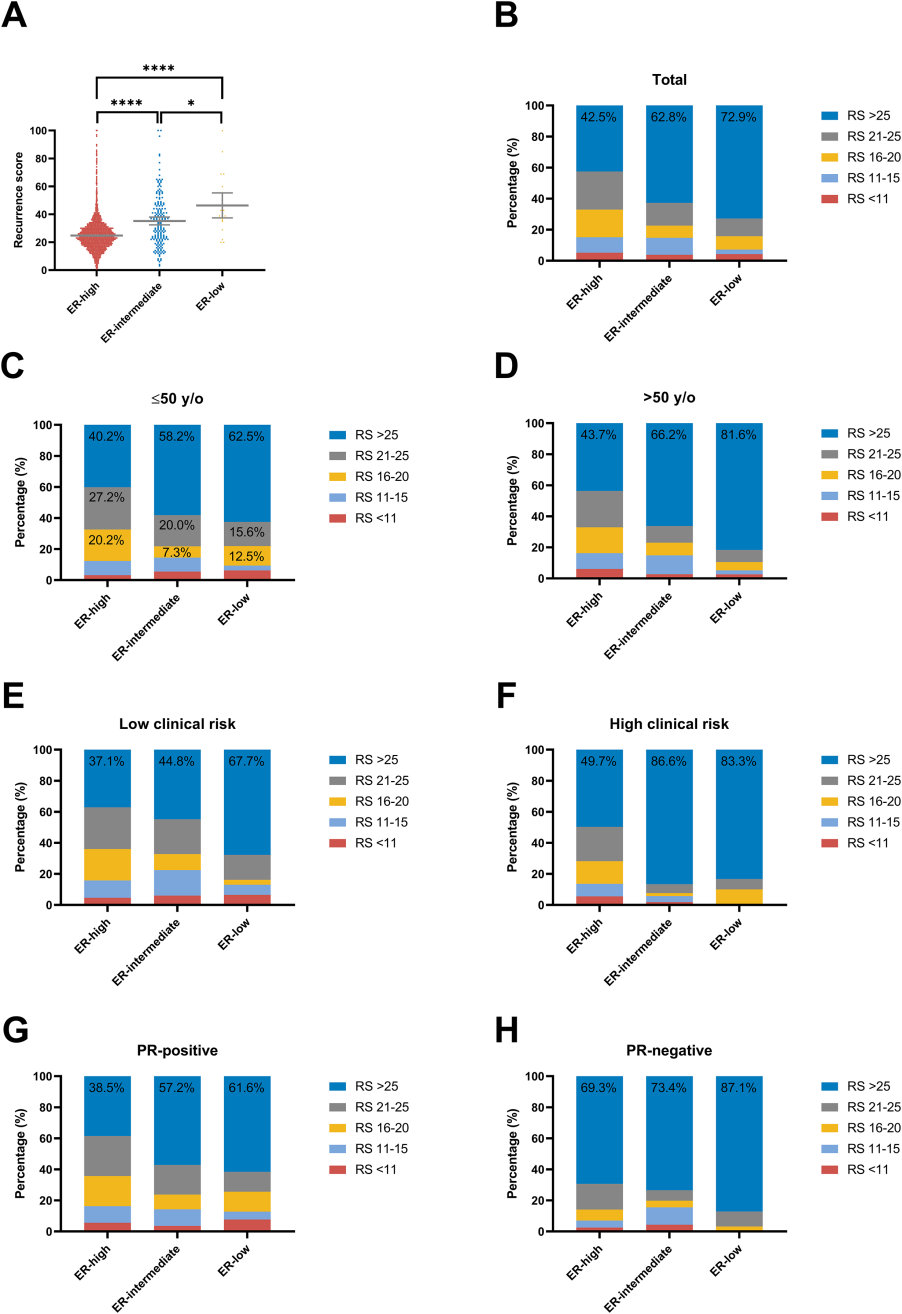
Distribution of the 21-gene RS in breast cancer patients with different ER levels. **(A)** In all, 21-gene RS as a continuous variable (Kruskal-Wallis test *P* < 0.001); The *P* values of multiple comparisons between groups were corrected using the Bonferroni method. 21-gene RS as a categorical variable in all patients **(B)** Chi-square test *P* < 0.001), patients aged ≤50 years **(C)** Chi-square test *P* = 0.007) and those aged >50 years **(D)** Chi-square test *P* < 0.001), patients with low **(E)** Chi-square test *P* = 0.008) or high clinical risk **(F)** Chi-square test *P* < 0.001), patients with PR-positive **(G)** Chi-square test *P* = 0.003) or PR-negative diseases **(H)** Chi-square test *P* = 0.127). *: P < 0.05; ****: P < 0.0001.

The mean 21-gene RS was 24 (IQR 19-30) in the ER-high group, 32 (IQR 21-44) in the ER-intermediate group, and 39 (IQR 24-56) in the ER-low group (*P* < 0.001, **Table 1**; **Figure 1A**). For patients with ER-high diseases, the proportions of RS <11, RS 11-25, and RS >25 were 5.2%, 52.3%, and 42.5%; for patients with ER-intermediate and ER-low diseases, the proportions were 3.9%, 33.3%, 62.8% and 4.2%, 22.9%, 72.9% respectively. The detailed distribution of 21-gene RS across categories (<11, 11-15, 16-20, 21-25, >25) among patients with different ER levels is illustrated in **Figure 1B** and **Supplementary Table 1**. The results of subgroup analysis according to age, clinical risk and PR status accorded with those in the entire population (**Figures 1C-H**). Of note, 62.5% patients aged 50 years or younger, 83.3% patients with high clinical risk, and 87.1% with PR-negative tumors had RS >25 in the ER-low group. Moreover, 12.5% had RS 16–20 and 15.6% had RS 21–25 among younger patients, collectively resulting in 90.6% having RS >15.

### Correlation between gene expression profiling and ER level

**Figure 2:**
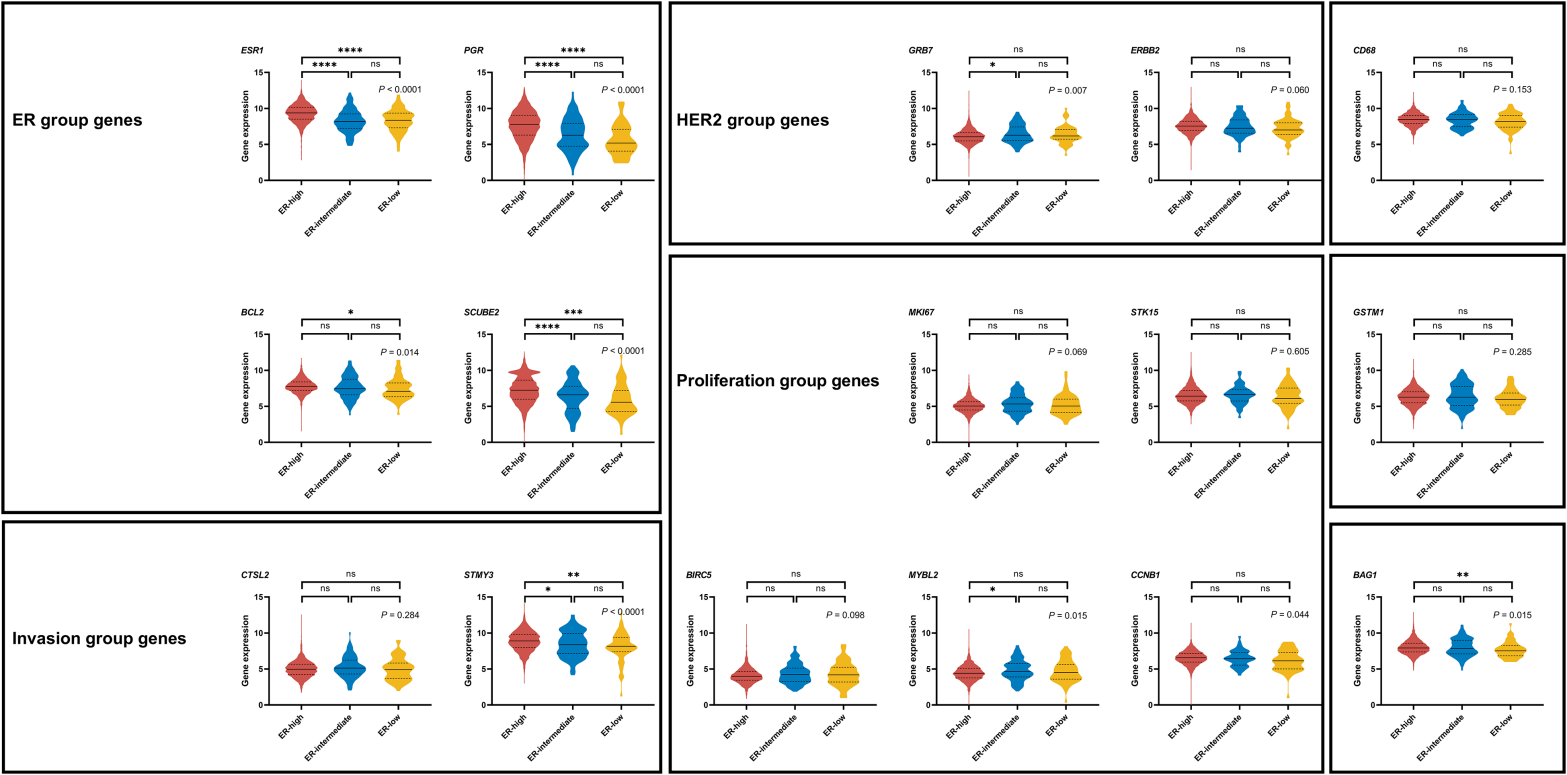
Individual gene expression levels of the 16 cancer genes from the 21-gene RS in breast cancer patients with different ER levels. . ns: not significant (*P* ≥ 0.05); *: *P* < 0.05; **: *P* < 0.01; ***: *P* < 0.001; ****: *P* < 0.0001.

As shown in **Figure 2**, we compared the expression levels of 16 functional genes among patients with different ER levels. The levels of *ESR1* (*P* < 0.001), *PGR* (*P* < 0.001), *BCL2* (*P* = 0.035), *SCUBE2* (*P* < 0.001) from the ER module, *STMY3* (*P* = 0.001) from the invasion module, and *BAG1* (*P* = 0.008) were significantly lower in ER-low tumors than in ER-high tumors. In contrast, the expressions of genes from the HER2 and proliferation group, as well as *CD68* and *GSTM1*, were comparable among the three groups.

### Correlation between RS and individual modules according to different ER level

**Figure 3:**
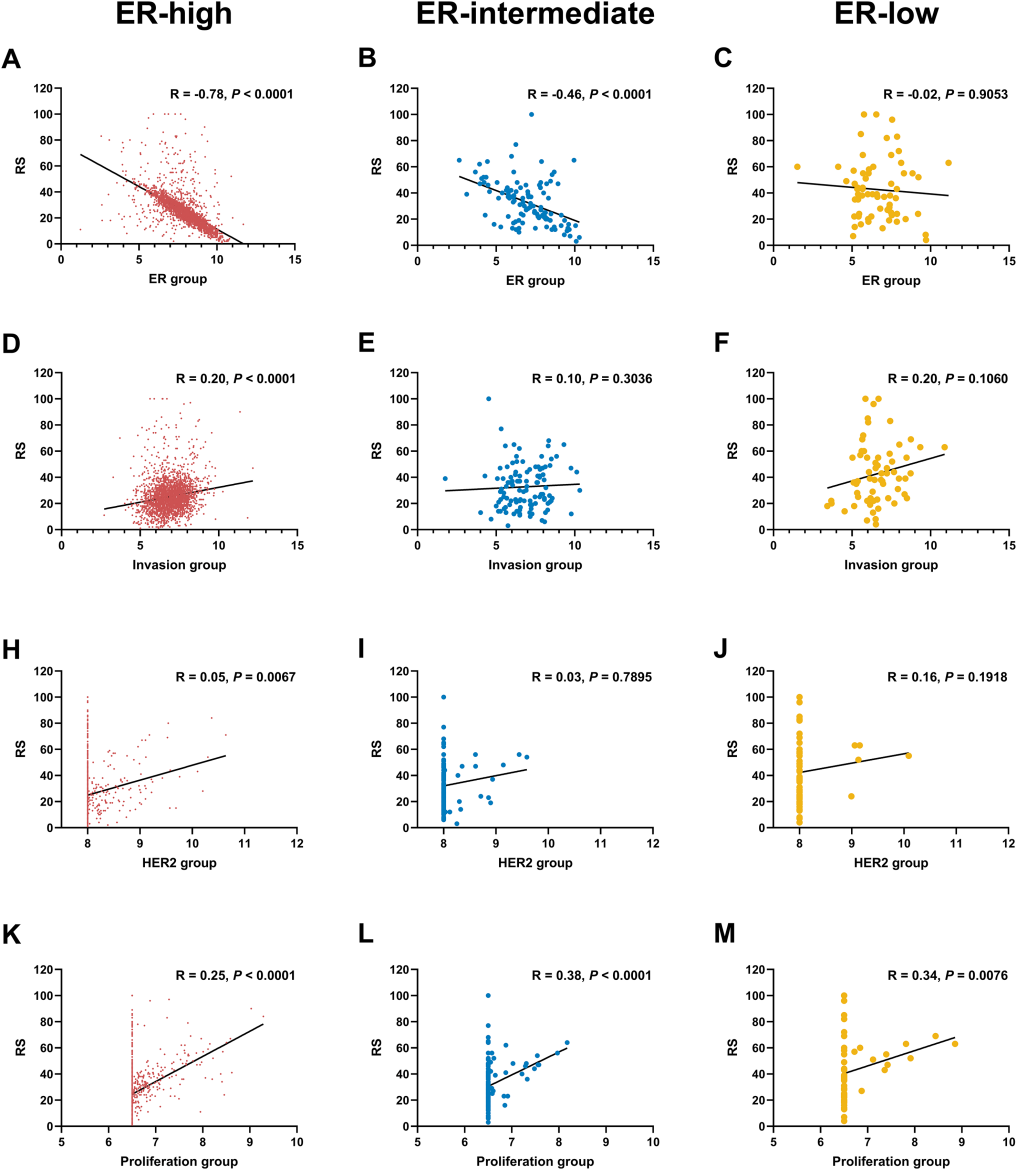
Correlation of RS scores with individual gene modules in breast cancer patients with different ER levels.

We further examined the relationship between 21-gene RS and its constituent modules in patients with different ER status (**Figure 3**). In patients with ER-high tumors, the ER module exhibited a moderate-to-strong negative correlation with RS (R = -0.78, 95% CI: -0.79 ~ -0.78, *P* < 0.001), whereas the correlations of proliferation (R = 0.25, 95% CI: 0.21 ~ 0.29, *P* < 0.001), invasion (R = 0.20, 95% CI: 0.16 ~ 0.23, *P* < 0.001), and HER2 (R = 0.05, 95% CI: 0.01 ~ 0.09, *P* = 0.0067) modules with RS were weak. In patients with ER-intermediate tumors, the ER (R = -0.46, 95% CI: -0.60 ~ -0.29, *P* < 0.001) and proliferation (R = 0.38, 95% CI: 0.20 ~ 0.54, *P* < 0.001) modules demonstrated modest correlation with RS, while the invasion (R = 0.10, 95% CI: -0.10 ~ 0.28, *P* = 0.3036) and HER2 (R = 0.05, 95% CI: -0.17 ~ 0.22, *P* = 0.7895) modules showed no significant correlations. In patients with ER-low diseases, only the proliferation module demonstrated a modest positive correlation with RS (R = 0.34, 95% CI: 0.09 ~ 0.55, *P* = 0.0076), while ER (R = -0.02, 95% CI: -0.27 ~ 0.24, *P* = 0.9053), invasion (R = 0.20, 95% CI: -0.05 ~ 0.43, *P* = 0.1060), and HER2 (R = 0.16, 95% CI: -0.09 ~ 0.40, *P* = 0.1918) modules had no significant correlations. Subgroup analysis by age, clinical risk and PR revealed similar trends and magnitudes of correlations between RS and its individual modules as observed in the entire cohort (**Supplementary Figure 1**-**6**).

### Contribution of individual modules to the variance of RS according to different ER level

**Figure 4:**
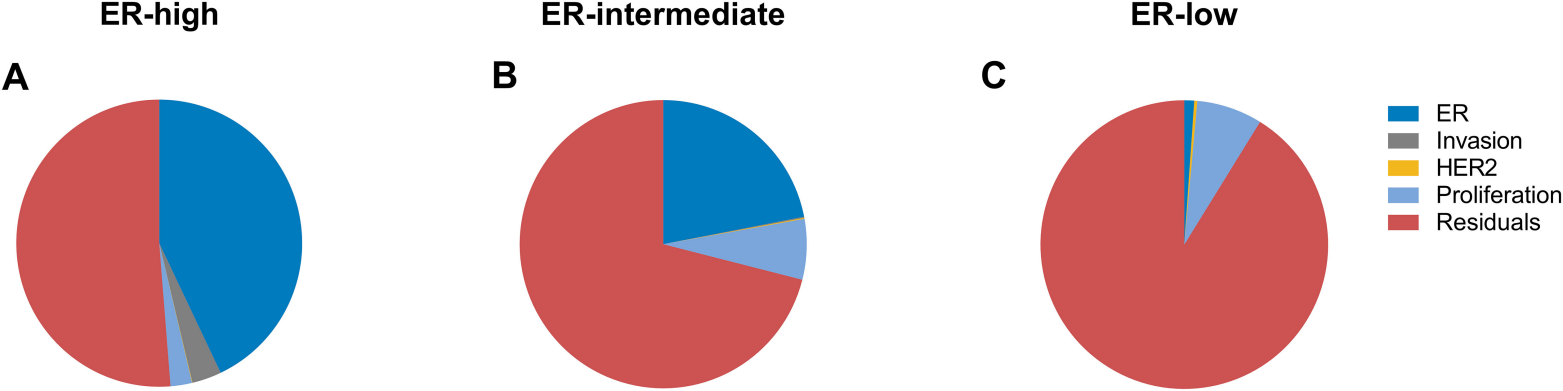
Variance of RS scores as accounted by individual gene modules in breast cancer patients with different ER levels.

Furthermore, variance analysis was performed to evaluate the contribution of each module to the variance of 21-gene RS (**Table 2** and **Figure 4**). Overall, the four gene modules explained 48.74% of the variance of RS in patients with ER-high tumors and 28.97% in patients with ER-intermediate diseases. However, the explained variance was substantially smaller in patients with ER-low tumors (8.83%). The distribution patterns of the variance contributions were also distinct among the three groups. In the ER-high group, the ER module was the primary contributor to the variance of RS (42.93%), while both the ER and proliferation modules contributed significantly in the ER-intermediate group (21.94% and 6.79%, respectively). In contrast, for patients with ER-low tumors, the proliferation module accounted for the largest proportion of RS variance (7.38%). Subgroup analyses based on age, clinical risk and PR status further elucidated that gene modules contributed less to RS among patients with ER-low tumors (**Supplementary Tables 4**-**6** and **Supplementary Figure 7**).

**Table 2:** Variance of RS scores as accounted by individual gene modules among different ER levels.

RS modules	ER-high		ER-intermediate		ER-low	
	Sum of squares	Variances explained (%)	Sum of squares	Variances explained (%)	Sum of squares	Variances explained (%)
ER	157584	42.93	5923	21.94	328	1.10
Invasion	12219	3.33	35	0.13	6	0.02
HER2 (unthresholded)	175	0.05	31	0.11	99	0.33
Proliferation (unthresholded)	8921	2.43	1833	6.79	2197	7.38
Residuals	188162	51.26	19177	71.03	27137	91.17

### Prognosis by ER level

A total of 4381 patients (92.2%) had intact survival data, with a median follow-up time of 53 (IQR 28-86) months. There were 227, 13, 18 DFS events and 34, 6, 11 OS events in patients with ER-high, intermediate, and low tumors, respectively. The estimated 5-year DFS rates were 94.3% (95% CI: 93.5%-95.2%) for the ER-high group, 92.2% (95% CI: 85.5%-95.9%) for the ER-intermediate group, and 83.2% (95% CI: 71.7%-90.3%) for the ER-low group (*P* < 0.001, **Supplementary Figure 8A**). Survival curves in patients with different ER levels according to RS are summarized in **Supplementary Figure 9** (*P* for interaction = 0.029). The corresponding 5-year OS rates were 99.4% (95% CI: 99.1%-99.6%), 96.5% (95% CI: 90.9%-98.7%) and 90.8% (95% CI: 80.8%-95.8%), respectively (*P* < 0.001, **Supplementary Figure 8B**). The results of univariate survival analysis for DFS and OS are summarized in **Supplementary Table 5**. In multivariate regression models, ER level was identified as an independent prognostic factor for both DFS (*P* = 0.008) and OS (*P* < 0.001; **Supplementary Table 6**). Patients in the ER-low group exhibited significantly higher risk of recurrence or death (HR for DFS 2.18, 95% CI: 1.33-3.57; HR for OS 10.42, 95% CI: 5.06-21.48).

## Discussion

Our findings indicate that ER-low, HER2-negative early breast cancer exhibits distinct biological features compared with ER-high tumors. The 21-gene RS was higher in patients with ER-low tumors and it was only moderately correlated with proliferation features, with minimal contribution from the four gene modules. No definitive conclusions can be drawn regarding the clinical utility of RS assay in this population before further clinical validation.

Previous researches have demonstrated that ER-low, HER2-negative breast cancer behaves similarly to TNBC (1–8, 27, 28). For example, a Swedish population-based cohort study of 5655 patients concluded that ER-low breast cancer had comparable characteristics and overall survival to TNBC (27). Another study enrolling 2765 patients from two large cohorts of neoadjuvant clinical trials (GeparQuinto and GeparSepto) revealed that HR-low tumors showed high pathological complete response rate and poor survival like TNBC (28). Likewise, this study revealed distinct clinical and pathological features of ER-low breast cancer, including a younger patient age, higher tumor grade and burden, increased proliferation rate and lower co-expression of hormone receptors, as well as a poorer prognosis. These characteristics suggest that ER-low breast cancer shares more similarities with TNBC than with typical ER-positive breast cancer, challenging the application of 21-gene assay in this subgroup.

The utility of the 21-gene assay in ER-low breast cancer has rarely been reported. Our findings showed that the mean 21-gene RS was significantly higher in ER-low patients compared to those with higher ER expression. Higgins and colleagues analyzed 689 ER-low breast cancer patients from the NCDB and reported that 67% had an RS >25 (29). Another smaller research by Loggie et al. suggested that 92.6% had an RS >25. In the present cohort, 72.9% ER-low, HER2-negative patients had an RS >25 (30). Along with RS, age and clinical risk are important factors when making decisions about adjuvant chemotherapy (22, 31, 32). We hence performed subgroup analyses according to different age, clinical risk as well as PR status of the patients, and further observed that this trend was particularly pronounced in younger patients, with 90.6% having RS >15; and those with high clinical risk or PR-negative tumors, with 83.3% or 87.1% having RS >25, respectively. Given that most ER-low patients may derive benefit from adjuvant chemotherapy, the clinical incremental value of RS testing in this population appears limited, particularly in high-risk cases. These observations remain exploratory and require confirmation.

Recent molecular subtyping of ER-low tumors has revealed that this subgroup is predominantly basal-like (29, 33, 34). We found that probably due to their intrinsic basal-like nature, the molecular drivers of RS in ER-low tumors were questionable. The gene expression analysis revealed that ER-low tumors had significantly lower expression of ER module genes (*ESR1*, *PGR*, *BCL2*, *SCUBE2*) compared to ER-high tumors. Correlation analysis indicated that the proliferation module rather than the ER-signaling genes demonstrated a modest correlation with RS in ER-low patients. Moreover, to objectively evaluate the contribution of the molecular features to the RS score, how much individual modules explained RS’s variance was also assessed. In ER-high tumors, ER module accounted for 42.93% of RS variance, which was in line with previous reports based on patients from the ATAC trial (35). In contrast, the overall contribution of the four modules to RS variance was minimal in ER-low tumors (totally 8.83%, 7.38% by proliferation module), leaving non-explained residues of 91.17%. Given the low explanatory power of the gene modules for RS variance, the current RS assay has limited molecular mechanistic basis in ER-low breast cancer. Development of more tailored prognostic tools is warranted for this subgroup. Notably, linear variance decomposition using aggregated module scores as surrogates may underestimate the true explainable variance of molecular features, particularly in ER-low tumors. Moreover, there is no uniform standard for the degree of contribution of a certain factor to the model. Therefore, the variance decomposition results were exploratory only and should be interpreted with caution.

More recently, neoadjuvant immunotherapy has represented a major advancement for the treatment of TNBC. Emerging data have demonstrated inspiring effects of immunotherapy among ER-low tumors (36, 37). For example, patients with ER-positive breast cancer receiving neoadjuvant chemotherapy and immunotherapy achieved relatively low percentage of pathological complete response (pCR, 24.3% in KEYNOTE-756 study (38) and 24.5% in CheckMate-7FL study (39)), but those with ER-low tumors had significantly higher pCR rates (55.9% and 55.6%, respectively). Moreover, a French real-world cohort revealed that 75% of 114 ER-low breast cancer patients achieved pCR with neoadjuvant pembrolizumab and chemotherapy (36), which was comparable to that observed in TNBC (64.8% in KEYNOTE-522 study (40)). Further investigations into the intrinsic molecular subtypes, tumor immune microenvironment (such as CD8/FOXP3 ratio, tumor-infiltrating lymphocyte density, and PD-L1 positivity rates), and immune-related gene expression profiles of ER-low breast tumors are warranted. Such studies could elucidate the underlying biology of this entity and inform the development of more tailored therapeutic strategies (34).

Despite the comprehensive analysis provided by this study, several limitations should be acknowledged. First, the retrospective, single-center design inherently introduces potential biases in data collection and patient selection, especially concerning which patients received 21-gene testing. This limits generalizability and underscores the need for validation in prospective, multi-center cohorts. Second, the number of ER-low breast cancer patients was relatively small (n=70, 1.5%), likely due to both the intrinsically low prevalence of this subtype and the limited application of the 21-gene assay in their clinical management. More importantly, subgroup analyses including survival results were based on a very limited number of patients or events, which raises concerns regarding statistical stability, model overfitting, and the reliability of effect estimates. Therefore, these findings should be interpreted with caution, and further studies with larger cohorts and more events are needed to validate the prognostic and predictive value of the 21-gene assay in ER-low tumors. Third, our study focused primarily on the 21-gene assay and did not assess potential cross-talk or interactions with other genes or molecular pathways, an area that requires deeper investigation.

In conclusion, our study demonstrates that the 21-gene RS was higher in ER-low breast cancer patients, particularly among younger individuals or those with high clinical risk or PR-negative diseases. In ER-low tumors, RS was only moderately correlated with the proliferation module and each of the four module components contributed little to its variance. Given the small sample size of the ER-low subgroup and few survival events, all results in this subgroup are exploratory and hypothesis-generating only.

## Data Availability

The raw data supporting the conclusions of this article will be made available by the authors, without undue reservation.
