# Supplementary material for: Distribution and molecular drivers of the 21-gene recurrence score in early breast cancer with low, intermediate, and high estrogen receptor expression
Source: Front Immunol. 2026 Apr 30;17:1653477. doi: 10.3389/fimmu.2026.1653477 (PMC13171759; doi:10.3389/fimmu.2026.1653477)
Supplement: Supplementary file 1 [file Table1.docx]

**Distribution and molecular drivers of 21-gene recurrence score in early breast cancer with low, intermediate and high estrogen receptor expression**

**Authors:**

Shuai Li ^1^, Jiahui Huang ^1^, Yifei Zhu ^1^, Jin Hong ^1^, Siji Zhu ^1^, Weiqi Gao ^1^, Ou Huang ^1^, Jianrong He ^1^, Weiguo Chen ^1^, Yafen Li ^1^, Xiaosong Chen ^1^, Kunwei Shen ^1^*, Jiayi Wu ^1^*

**Affiliation:**

^1^ Department of General Surgery, Comprehensive Breast Health Center, Ruijin Hospital, Shanghai Jiao Tong University School of Medicine, Shanghai, China

**Correspondence to:**

Jiayi Wu, 22nd Floor, 197 Ruijin Er Road, Shanghai 200025, China. E-mail: pinkscorpio@163.com

Kunwei Shen, 22nd Floor, 197 Ruijin Er Road, Shanghai 200025, China. E-mail: [kwshen@medmail.com.cn](mailto:kwshen@medmail.com.cn)

**Running Title**:

S Li et al: 21-gene in ER-low EBC

**Supplementary Table S1** Distribution of 21-gene RS by ER level.

| Characteristics | Total | ER-high | ER-intermediate | ER-low | *P* value |
| --- | --- | --- | --- | --- | --- |
| **Total** |  |  |  |  | **<0.001** |
| < 11 | 243 (5.1) | 235 (5.2) | 5 (3.9) | 3 (4.2) |  |
| 11-15 | 467 (9.8) | 451 (9.9) | 14 (10.9) | 2 (2.9) |  |
| 16-20 | 826 (17.4) | 810 (17.8) | 10 (7.7) | 6 (8.6) |  |
| 21-25 | 1149 (24.2) | 1122 (24.6) | 19 (14.7) | 8 (11.4) |  |
| > 25 | 2069 (43.5) | 1937 (42.5) | 81 (62.8) | 51 (72.9) |  |
| **≤50 y/o** |  |  |  |  | **0.017** |
| < 11 | 52 (3.3) | 47 (3.2) | 3 (5.5) | 2 (6.3) |  |
| 11-15 | 143 (9.1) | 137 (9.2) | 5 (9.0) | 1 (3.1) |  |
| 16-20 | 309 (19.6) | 301 (20.2) | 4 (7.3) | 4 (12.5) |  |
| 21-25 | 420 (26.7) | 404 (27.2) | 11 (20.0) | 5 (15.6) |  |
| > 25 | 650 (41.3) | 598 (40.2) | 32 (58.2) | 20 (62.5) |  |
| **>50 y/o** |  |  |  |  | **<0.001** |
| < 11 | 191 (6.0) | 188 (6.1) | 2 (2.7) | 1 (2.6) |  |
| 11-15 | 324 (10.2) | 314 (10.2) | 9 (12.2) | 1 (2.6) |  |
| 16-20 | 517 (16.3) | 509 (16.6) | 6 (8.1) | 2 (5.3) |  |
| 21-25 | 729 (22.9) | 718 (23.4) | 8 (10.8) | 3 (7.9) |  |
| > 25 | 1419 (44.6) | 1339 (43.7) | 49 (66.2) | 31 (81.6) |  |
| **Low clinical risk** |  |  |  |  | **0.008** |
| < 11 | 112 (4.7) | 106 (4.6) | 4 (6.0) | 2 (6.5) |  |
| 11-15 | 271 (11.4) | 258 (11.2) | 11 (16.4) | 2 (6.5) |  |
| 16-20 | 473 (19.7) | 465 (20.2) | 7 (10.4) | 1 (3.2) |  |
| 21-25 | 639 (26.6) | 619 (26.9) | 15 (22.4) | 5 (16.1) |  |
| > 25 | 903 (37.6) | 852 (37.1) | 30 (44.8) | 21 (67.7) |  |
| **High** **clinical risk** |  |  |  |  | **<0.001** |
| < 11 | 106 (5.3) | 105 (5.5) | 1 (1.9) | 0 (0.0) |  |
| 11-15 | 158 (7.9) | 156 (8.1) | 2 (3.8) | 0 (0.0) |  |
| 16-20 | 285 (14.2) | 281 (14.6) | 1 (1.9) | 3 (10.0) |  |
| 21-25 | 430 (21.5) | 425 (22.1) | 3 (5.8) | 2 (6.7) |  |
| > 25 | 1025 (51.1) | 955 (47.1) | 45 (86.6) | 25 (83.3) |  |
| **PR-positive** |  |  |  |  | **0.003** |
| < 11 | 226 (5.5) | 220 (5.6) | 3 (3.6) | 3 (7.7) |  |
| 11-15 | 435 (10.7) | 424 (10.7) | 9 (10.7) | 2 (5.1) |  |
| 16-20 | 781 (19.1) | 768 (19.4) | 8 (9.5) | 5 (12.8) |  |
| 21-25 | 1044 (25.6) | 1023 (25.8) | 16 (19.0) | 5 (12.8) |  |
| > 25 | 1597 (39.1) | 1525 (38.5) | 48 (57.2) | 24 (61.6) |  |
| **PR-negative** |  |  |  |  | **0.127** |
| < 11 | 17 (2.5) | 15 (2.5) | 2 (4.4) | 0 (0.0) |  |
| 11-15 | 32 (4.8) | 27 (4.5) | 5 (11.1) | 0 (0.0) |  |
| 16-20 | 45 (6.7) | 42 (7.1) | 2 (4.4) | 1 (3.2) |  |
| 21-25 | 105 (15.6) | 99 (16.6) | 3 (6.7) | 3 (9.7) |  |
| > 25 | 472 (70.4) | 412 (69.3) | 33 (73.4) | 27 (87.1) |  |

**Supplementary Table S2** Variance of RS scores as accounted by individual gene modules among different ER levels and subgroups by age.

| **RS modules** | **ER-high** | |  | **ER-intermediate** | |  | **ER-low** | |
| --- | --- | --- | --- | --- | --- | --- | --- | --- |
|  | Sum of squares | Variances  explained (%) |  | Sum of squares | Variances  explained (%) |  | Sum of squares | Variances  explained (%) |
| ≤50 y/o |  |  |  |  |  |  |  |  |
| ER | 38925 | 36.50 |  | 3365 | 29.50 |  | 1814 | 12.52 |
| Invasion | 4507 | 4.23 |  | 393 | 3.44 |  | 119 | 0.82 |
| HER2 (unthresholded) | 24 | 0.02 |  | 127 | 1.11 |  | 143 | 0.99 |
| Proliferation (unthresholded) | 2996 | 2.81 |  | 575 | 5.04 |  | 1262 | 8.71 |
| Residuals | 60196 | 56.44 |  | 6947 | 60.90 |  | 11152 | 76.97 |
| >50 y/o |  |  |  |  |  |  |  |  |
| ER | 118008 | 46.14 |  | 1701 | 11.72 |  | 261 | 1.90 |
| Invasion | 7673 | 3.00 |  | 207 | 1.43 |  | 40 | 0.29 |
| HER2 (unthresholded) | 242 | 0.09 |  | 130 | 0.90 |  | 61 | 0.44 |
| Proliferation (unthresholded) | 5077 | 1.99 |  | 1343 | 9.25 |  | 180 | 1.31 |
| Residuals | 124763 | 48.78 |  | 11137 | 76.71 |  | 13205 | 96.06 |

**Supplementary Table S3** Variance of RS scores as accounted by individual gene modules among different ER levels and subgroups by clinical risk.

| **RS modules** | **ER-high** | |  | **ER-intermediate** | |  | **ER-low** | |
| --- | --- | --- | --- | --- | --- | --- | --- | --- |
|  | Sum of squares | Variances  explained (%) |  | Sum of squares | Variances  explained (%) |  | Sum of squares | Variances  explained (%) |
| Low clinical risk |  |  |  |  |  |  |  |  |
| ER | 68446 | 44.08 |  | 2255 | 21.62 |  | 13 | 0.11 |
| Invasion | 7761 | 5.00 |  | 3 | 0.03 |  | 0 | 0.00 |
| HER2 (unthresholded) | 18 | 0.01 |  | 8 | 0.07 |  | 11 | 0.09 |
| Proliferation (unthresholded) | 2258 | 1.45 |  | 627 | 6.01 |  | 111 | 0.92 |
| Residuals | 76802 | 49.46 |  | 7538 | 72.27 |  | 11942 | 98.88 |
| High clinical risk |  |  |  |  |  |  |  |  |
| ER | 76089 | 43.37 |  | 1236 | 11.13 |  | 1 | 0.01 |
| Invasion | 3636 | 2.07 |  | 7 | 0.06 |  | 5 | 0.04 |
| HER2 (unthresholded) | 135 | 0.08 |  | 182 | 1.64 |  | 786 | 5.90 |
| Proliferation (unthresholded) | 4657 | 2.65 |  | 224 | 2.02 |  | 1450 | 10.88 |
| Residuals | 90912 | 51.82 |  | 9449 | 85.14 |  | 11084 | 83.17 |

**Supplementary Table S4** Variance of RS scores as accounted by individual gene modules among different ER levels and subgroups by PR status.

| **RS modules** | **ER-high** | |  | **ER-intermediate** | |  | **ER-low** | |
| --- | --- | --- | --- | --- | --- | --- | --- | --- |
|  | Sum of squares | Variances  explained (%) |  | Sum of squares | Variances  explained (%) |  | Sum of squares | Variances  explained (%) |
| PR-positive |  |  |  |  |  |  |  |  |
| ER | 115415 | 41.76 |  | 3562 | 24.78 |  | 706 | 4.03 |
| Invasion | 11400 | 4.12 |  | 780 | 5.43 |  | 172 | 0.98 |
| HER2 (unthresholded) | 308 | 0.11 |  | 34 | 0.24 |  | 64 | 0.37 |
| Proliferation (unthresholded) | 6147 | 2.22 |  | 462 | 3.21 |  | 2692 | 15.37 |
| Residuals | 143113 | 51.78 |  | 9539 | 66.35 |  | 13880 | 79.25 |
| PR-negative |  |  |  |  |  |  |  |  |
| ER | 26651 | 38.97 |  | 485 | 4.39 |  | 48 | 0.43 |
| Invasion | 1322 | 1.93 |  | 1127 | 10.20 |  | 4 | 0.03 |
| HER2 (unthresholded) | 365 | 0.53 |  | 0 | 0 |  | 358 | 3.17 |
| Proliferation (unthresholded) | 818 | 1.20 |  | 2258 | 20.44 |  | 263 | 2.33 |
| Residuals | 39237 | 57.37 |  | 7176 | 64.97 |  | 10630 | 94.05 |

**Supplementary Table S5** Univariate analysis of prognostic factors affecting DFS and OS

| Characteristics | *P* value | |
| --- | --- | --- |
|  | DFS | OS |
| Age (≤ 50 y/o *vs.* > 50 y/o) | 0.155 | 0.073 |
| Histology type (IDC *vs.* Non-IDC) | 0.492 | 0.709 |
| Tumor size (≤ 2.0 cm *vs.* > 2.0 cm) | **<0.001** | **<0.001** |
| ALN status (Negative *vs.* Positive) | 0.418 | 0.841 |
| Histological grade (Ⅰ *vs.* Ⅱ *vs.* Ⅲ *vs.* NA) | **<0.001** | **0.007** |
| PR status (Negative *vs.* Positive) | **0.004** | **0.040** |
| HER2 status (- *vs.* +/++) | 0.584 | 0.282 |
| Ki67 level (≤ 20% *vs.* > 20%) | **<0.001** | 0.295 |
| 21-gene RS (< 11 *vs.* 11-25 *vs.* > 25) | **<0.001** | **0.003** |
| ER level (High *vs.* Intermediate *vs.* Low) | **<0.001** | **<0.001** |

Abbreviations: ALN, axillary lymph node; DFS, disease-free survival; ER, estrogen receptor; HER2, human epidermal growth factor receptor-2; IDC, invasive ductal carcinoma; NA, not available; OS, overall survival; PR, progesterone receptor; RS: recurrence score; y/o, years old.

The significant *P* values are in bold.

**Supplementary Table S6** Multivariate Cox proportional regression analysis of prognostic factors affecting DFS and OS

| Characteristics | **DFS** | |  | **OS** | |
| --- | --- | --- | --- | --- | --- |
|  | *HR* (95% CI) | *P* value |  | *HR* (95% CI) | *P* value |
| **Age (y/o)** |  | 0.220 |  |  | **0.011** |
| ≤ 50 | 1.00 |  |  | 1.00 |  |
| > 50 | 0.86 (0.67-1.10) |  |  | 2.19 (1.19-4.02) |  |
| **Histology type** |  | 0.252 |  |  | 0.381 |
| IDC | 1.00 |  |  | 1.00 |  |
| Non-IDC | 1.42 (0.78-2.60) |  |  | 0.52 (0.12-2.27) |  |
| **Tumor size** |  | **<0.001** |  |  | **0.011** |
| ≤ 2.0 cm | 1.00 |  |  | 1.00 |  |
| > 2.0 cm | 1.58 (1.23-2.03) |  |  | 2.06 (1.18-3.60) |  |
| **ALN status** |  | 0.835 |  |  | 0.575 |
| Negative | 1.00 |  |  | 1.00 |  |
| Positive | 1.04 (0.74-1.45) |  |  | 0.79 (0.34-1.81) |  |
| **Histological grade** |  | **0.009** |  |  | 0.150 |
| Ⅰ/II | 1.00 |  |  | 1.00 |  |
| Ⅲ | 1.57 (1.16-2.12) |  |  | 1.69 (0.88-3.24) |  |
| NA | 1.32 (0.93-1.89) |  |  | 1.85 (0.88-3.90) |  |
| **PR status** |  | 0.202 |  |  | 0.725 |
| Negative | 1.00 |  |  | 1.00 |  |
| Positive | 1.22 (0.90-1.66) |  |  | 0.89 (0.45-1.55) |  |
| **HER2 status** |  | 0.641 |  |  | 0.578 |
| - | 1.00 |  |  | 0.83 (0.42-1.62) |  |
| +/++ | 0.93 (0.68-1.27) |  |  |  |  |
| **Ki67 level** |  | **0.011** |  |  | 0.494 |
| ≤ 20% | 1.00 |  |  | 1.00 |  |
| > 20% | 1.42 (1.08-1.87) |  |  | 0.80 (0.43-1.50) |  |
| **21-gene RS** |  | **0.042** |  |  | 0.086 |
| < 11 | 1.00 |  |  | 1.00 |  |
| 11-25 | 1.12 (0.59-2.16) |  |  | 1.90 (0.25-14.39) |  |
| > 25 | 1.54 (0.81-2.95) |  |  | 3.54 (0.48-26.05) |  |
| **ER level** |  | **0.008** |  |  | **<0.001** |
| High | 1.00 |  |  | 1.00 |  |
| Intermediate | 1.07 (0.61-1.88) |  |  | 3.87 (1.60-9.41) |  |
| Low | 2.18 (1.33-3.57) |  |  | 10.42 (5.06-21.48) |  |

Abbreviations: ALN, axillary lymph node; DFS, disease-free survival; ER, estrogen receptor; HER2, human epidermal growth factor receptor-2; IDC, invasive ductal carcinoma; NA, not available; OS, overall survival; PR, progesterone receptor; RS: recurrence score; y/o, years old.

The significant *P* values are in bold.





**Supplementary Figure S1 Correlation of RS scores with individual gene modules in breast cancer patients ≤50 y/o with different ER levels.**





**Supplementary Figure S2 Correlation of RS scores with individual gene modules in breast cancer patients >50 y/o with different ER levels.**

**

**

**Supplementary Figure S3 Correlation of RS scores with individual gene modules in breast cancer patients with low clinical risk and different ER levels.**





**Supplementary Figure S4 Correlation of RS scores with individual gene modules in breast cancer patients with high clinical risk and different ER levels.**





**Supplementary Figure S5 Correlation of RS scores with individual gene modules in breast cancer patients with PR-positive tumors and different ER levels.**

**

**

**Supplementary Figure S6 Correlation of RS scores with individual gene modules in breast cancer patients with PR-negative tumors and different ER levels.**





**Supplementary Figure S7 Variance of RS scores as accounted by individual gene modules in breast cancer with different ER levels and subgroups.**





**Supplementary Figure S8** **Kaplan-Meier curves of DFS and OS by ER level.** (A) The estimated 5-year DFS rates for the ER-high group, the ER-intermediate group and the ER-low group were 94.3%, 92.2% and 83.2%, respectively (*P* < 0.001). The DFS events were 227, 13, 18, respectively. (B) The estimated 5-year OS rates for the three groups were 99.4%, 96.5% and 90.8%, respectively (*P* < 0.001). The OS events were 34, 6, 11, respectively.





**Supplementary Figure S9** **Kaplan-Meier curves of DFS by RS in patients with different ER levels.** There was significant interaction between RS and ER level (*P* = 0.029).
